# *In vitro* study of ivermectin efficiency against the cattle tick, *Rhipicephalus* (*Boophilus*) *annulatus*, among cattle herds in El-Beheira, Egypt

**DOI:** 10.14202/vetworld.2019.1319-1326

**Published:** 2019-08-25

**Authors:** Gaber E. Batiha, Ali H. El-Far, Amany A. El-Mleeh, Abdelwahab A. Alsenosy, Eman K. Abdelsamei, Mohamed M. Abdel-Daim, Yasser S. El-Sayed, Hazem M. Shaheen

**Affiliations:** 1 Department of Pharmacology, Faculty of Veterinary Medicine, Damanhour University, Egypt; 2 Department of Biochemistry, Faculty of Veterinary Medicine, Damanhour University, Egypt; 3 Department of Pharmacology, Faculty of Veterinary Medicine, Menoufia University, Egypt; 4 Department of Parasitology, Faculty of Veterinary Medicine, Menoufia University, Egypt; 5 Department of Pharmacology, Faculty of Veterinary Medicine, Suez Canal University, Ismailia 41522, Egypt; 6 Department of Veterinary Forensic Medicine and Toxicology, Faculty of Veterinary Medicine, Damanhour University, Egypt

**Keywords:** acaricidal, glutathione S-transferase, ivermectin, malondialdehyde, *Rhipicephalus* (*Boophilus*) *annulatus*, γ-aminobutyric acid

## Abstract

**Background and Aim:**

Ivermectin (IVM) has been used in veterinary practice to control different parasitic infestations over the past two decades. This study aimed to re-assess the acaricidal effects of IVM, as well as to evaluate its efficacy against *Rhipicephalus* (*Boophilus) annulatus* by determining the mortality rate, γ-aminobutyric acid (GABA) level, and oxidative/antioxidative homeostasis (malondialdehyde [MDA] levels and glutathione S-transferase [GST] activities).

**Materials and Methods:**

Adult female *Rhipicephalus* (*Boophilus) annulatus* were picked from cattle farms in El-Beheira Governorate, Egypt. Ticks were equally allocated to seven experimental groups to assess the acaricidal potential of IVM chemotherapeutics in controlling *R*. (*B*.) *annulatus*. IVM was prepared at three concentrations (11.43, 17.14, and 34.28 µM of IVM).

**Results:**

Mortality rate was calculated among the treated ticks. In addition, GABA, GST, and MDA biomarker levels were monitored. The data revealed a noticeable change in GST activity, a detoxification enzyme found in *R*. (*B*.) *annulatus*, through a critical elevation in mortality percentage.

**Conclusion:**

IVM-induced potent acaricidal effects against *R*. (*B*.) *annulatus* by repressing GST activity for the initial 24 h after treatment. Collectively, this paper reports the efficacy of IVM in a field population of *R*. (*B*.) *annulatus* in Egypt.

## Introduction

Accurate estimation of losses due to ticks and tick-borne diseases (TTBDs) is difficult but important because they can have significant effects on livestock. Research has shown that an infestation of an average of 105 ticks on a crossbred Holstein-Zebu cow results in a 23% reduction in milk yield/day [1]. Losing about ¼ of the income from milk as a result of a tick burden has had a significant impact on livestock-dependent systems [1]. TTBDs ranks fourth among the major livestock infections in Egypt and are considered the most important arthropod-borne diseases of livestock [2].

*Rhipicephalus* (*Boophilus*) *annulatus* is a hard tick that is considered a biological vector for blood parasites such as *Babesia*, *Anaplasma*, and *Theileria* spp. Infestation of *R*. (*B*.) *annulatus* in cattle induces skin irritation and continuous rubbing that may lead to skin injuries [3]. *R*. (*B*.) *annulatus* population control has been implemented by the application of several classes of acaricides, including macrocyclic lactone (ML) derivatives such as ivermectin (IVM) [4,5]. IVM is composed of 22, 23-dihydroavermectin-B1a (80%) and 22, 23-dihydroavermectin-B1b (20%) [6], and is generally used for the control of endo- and ecto-parasites [7,8]. IVM paralyzes arthropods by the disrupting of γ-aminobutyric acid (GABA)-dependent neurotransmission, resulting in death due to failure of adherence to a host or extended cessation of feeding [6,8]. While IVM does not interfere with the GABA system necessary for enhancing salivary fluid secretion, it may interfere with other GABA systems in ticks [9].

Of late, interest in the re-assessment of acaricidal bioactivity has increased among researchers. For instance, Klafke *et al*. [10] assessed the efficacy of IVM among *Rhipicephalus microplus* populations, demonstrating that larval immersion tests could be an important method to determine IVM efficacy. In addition, studies have shown that the ML class of acaricides are effective against number of parasites [11-13], including the first demonstration of ML efficacy among ticks in Brazil [14]. Sensitivity to ML (doramectin, IVM, and moxidectin) varies as shown by less-sensitive *R. microplus* in Brazil and Mexico [10,15,16]. In Egypt, the efficacy of IVM on *R*. (*B*.) *annulatus* field populations has not received much attention. Both veterinarians and farmers have complained about unsuccessful IVM treatments under field conditions even after repeated doses.

This study aimed to re-assess the acaricidal effects of IVM, as well as to evaluate its efficacy against *R*. (*B*.) *annulatus* by determining the mortality rate, GABA level, and oxidative/antioxidative homeostasis (malondialdehyde [MDA] levels and glutathione S-transferase [GST] activities).

## Materials and Methods

### Ethics approval

The protocol was approved by the Committee of Local Experimental Animal Care of the Faculty of Veterinary Medicine, Damanhour University, Egypt (Ethical Issue: #VM1522/2010).

### Chemicals

IVM chemotherapeutics (IVM A; IVM 10 mg/mL and IVM B; IVM 10 mg plus clorsulon 100 mg/mL) were provided by the Central Agricultural Pesticide Laboratory, National Center for Agricultural Research, Ministry of Agriculture, Giza, Egypt. The GABA ELISA kit (MBS740443) was ordered from MyBioSource Company, USA. MDA and GST kits were purchased from Bio-Diagnostic Company, Egypt.

### *R. (B.) annulatus* preparation

**Figure-1:**
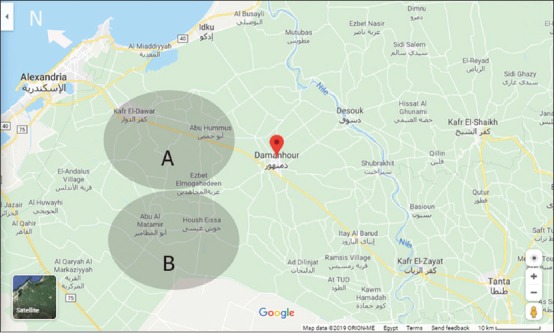
Map of the study regions (A and B), El-Beheira Governorate, Egypt [Source: Google map].

*R*. (*B*.) *annulatus* were manually harvested from well-defined and reliable cattle sources of El-Beheira Governorate (30.61°N-30.43°E), Egypt. The cattle, distributed between two locations (Figure-1), were not treated with acaricides for at least 50 days before collection of the *R*. (*B*.) *annulatus* samples. Active, mature, and engorged female *R*. (*B*.) *annulatus* were collected directly from animals from March 2015 to May 2015 and transported to the laboratory in humidified polystyrene boxes [17].

*R*. (*B*.) *annulatus* were identified under a stereomicroscope, according to the recommended identification marks [17-20]. Female *R*. (*B*.) *annulatus* had body lengths greater than 5 mm (no standard engorging female *R*. (*B*.) *annulatus* were ≥5 mm) and partially covered with scutum on their back, appearing as a dark brown pinpoint on the back.

The *R*. (*B*.) *annulatus* samples were transferred to the Pharmacology Research Laboratory, Faculty of Veterinary Medicine, Damanhour University, Egypt. The samples were assigned to seven experimental groups (*n*=90 each). The bioassays were conducted on the same day the *R*. (*B*.) *annulatus* were collected.

### Experimental design

IVM chemotherapeutics were mixed in pure ethanol to create an IVM stock solution. Ethanol (Et) was added to Triton X-100 (Tx) to create a 2% solution, which was then diluted in distilled water to a final concentration of Et+Tx of 1%. The IVM stock solution was added to the 1% Tx+Et to create a final concentration of 0.01% IVM. The concentrations of IVM in different immersion solutions were calculated in µM. The 1% Et+Tx solution was used as a control solution [15]. The tested IVM concentrations were modified and managed for the adult immersion test, as described previously [21,22].

*R*. (*B*.) *annulatus* were assigned to seven equal groups. Each group was replicated three times (30 per each replicate) and exposed to the testing chemotherapeutic, according to the principle discussed in a previous study [23]:

Control (Et+Tx 1%) (n=90); 30 *R*. (*B*.) *annulatus* per replicate immersed in Et+Tx 1%.

IVM A-I (n=90); 30 *R*. (*B*.) *annulatus* per replicate, immersed in 11.43 µM of IVM.

IVM A-II (n=90); 30 *R*. (*B*.) *annulatus* per replicate, immersed in 17.14 µM of IVM.

IVM A-III (n=90); 30 *R*. (*B*.) *annulatus* per replicate, immersed in 34.28 µM of IVM.

IVM B-I (n=90); 30 *R*. (*B*.) *annulatus* per replicate, immersed in 11.43 µM of IVM.

IVM B-II (n=90); 30 *R*. (*B*.) *annulatus* per replicate, immersed in 17.14 µM of IVM.

IVM B-III (n=90); 30 *R*. (*B*.) *annulatus* per replicate, immersed in 34.28 µM of IVM.

Approximately 20 mL of each treatment liquid was poured into 10 cm Petri dishes. *R*. (*B*.) *annulatus* were immersed in these treatment solutions for 10 min at 25°C. Then, the *R*. (*B*.) *annulatus* were removed from the solution and treated 3 times with a spray of freshly distilled water for 1 min. The vitality signs were observed every 10 min, and the numbers of dead and live *R*. (*B*.) *annulatus* were recorded after 0.5, 1, 3, 24, 48, and 72 h.

### Vitality signs

To screen the sensitivity of *R*. (*B*.) *annulatus* to the tested IVM chemotherapeutics, the medium was incubated with increasing doses of IVM A and IVM B (11.43, 17.14, and 34.28 µM) for 0.5, 1, 3, 24, 48, and 72 h. The mortality of *R*. (*B*.) *annulatus* was calculated and compared to the control.

The dead and live *R*. (*B*.) *annulatus* were observed through a stereomicroscope, and *R*. (*B*.) *annulatus* were considered dead when they did not react to either light exposure or a slight touch with blunt forceps after 5 min. *R*. (*B*.) *annulatus* movement and viability were physically observed and recorded by the authors.

The percent control due to treatment was calculated by a modification of Abbott’s formula [24]:


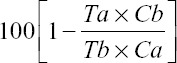


Where, *Ta* is the number of *R*. (*B*.) *annulatus* collected after treatment, *Tb* is the number of *R*. (*B*.) *annulatus* collected per sampling unit before treatment, *Ca* is the number of *R*. (*B*.) *annulatus* collected from the check plot after treatment of the test plots, and *Cb* is the number of *R*. (*B*.) *annulatus* collected from the check plot before treatment.

The above formula was used to calculate the reduction rate (or mortality rate) among *R*. (*B*.) *annulatus* population after the application of the tested acaricide. The corrected efficacy percentage was calculated only when the counts in the treated groups were significantly lower than the count in the control group.

### Preparation of tissue homogenate

Guts were dissected from the live *R*. (*B*.) *annulatus* at 0.5, 1, 3, 24, 48, and 72 h after treatment. Gut tissues were homogenized in 10 mL phosphate-buffered saline (pH, 7.4) by a homogenizer and centrifuged at 4000× *g* for 15 min. The clear supernatants were collected, and GABA, GST, and MDA levels were analyzed.

Folin phenol reagent was used to standardize the levels of GABA, GST, and MDA. The protein content of the collected supernatant was determined as per a previously described method [25].

### GABA assay

GABA levels were determined using an ELISA kit (MyBioSource, California, San Diego, USA) with an estimated sensitivity of 0.1 nM/mL from gradient standard curves.

### Spectrophotometric analysis of GST

A bio-diagnostic GST assay kit (Bio-Diagnostic Co., Giza, Egypt) was used to determine the activity of total GST by measuring the intensities of 1-chloro-2,4-dinitrobenzene (CDNB) conjugation with reduced glutathione at 340 nm [26]. The reaction mixture was created by combining 1 mL sodium phosphate buffer 0.1 M (pH 7.4), 0.1 mL GSH (9.2 mM), 0.1 mL CDNB (0.1 M), and 0.05 mL of the sample. The reaction was controlled by adding 0.1 mL of 5% trichloroacetic acid. The absorbance was determined at 340 nm after centrifugation at 3000× *g* for 5 min. The changes in absorbance were determined every 1 min for 3 min.

### Spectrophotometric analysis of MDA

MDA was analyzed in the homogenized tissues using the thiobarbituric acid (TBA) reactive substance assay [27]. MDA was reacted with TBA at 100°C in an acidic medium to form a pink-colored complex. The intensity of the complex was measured at 535 nm, and it was proportional to the degree of MDA-TBA formed.

### Statistical analysis

Microsoft Excel was used for two-way ANOVA and Student’s t-test analysis. Differences in the means were considered significant at p<0.05.

## Results

**Figure-2:**
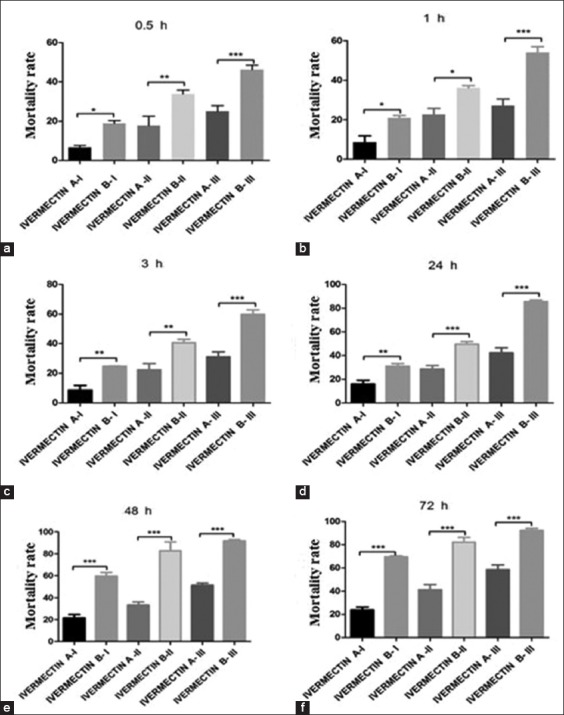
The mortality rates of treatment groups; ivermectin (IVM) A (IVM 10 mg/mL) and IVM B (IVM 10 mg plus clorsulon 100 mg/mL); groups were analyzed by two-way ANOVA. *p<0.05 and **p<0.01 and ***p<0.001; IVM A-I versus IVM B-I; IVM A-II versus IVM B-II and IVM A-III versus IVM B-III groups by an unpaired t-test.

IVM A and IVM B were used in this study to check acaricidal effects against *R*. (*B*.) *annulatus*, as presented in Figure-2. The mortality rate of ticks increased with exposure time. The effect of IVM B on *R*. (*B*.) *annulatus* survival was significantly higher than IVM A. Efficacy of IVM B increased with exposure time, with the highest mortality rate observed at 72 h. Mortality rates of IVM B (II and III) at 72 h were 82.22% and 92.33%, respectively. Efficacy of IVM A also increased with exposure time but was much less effective than IVM B. The highest mortality rate for IVM A was observed at 72 h. Mortality rates of IVM A III at 48 h and 72 h were 51.25% and 58.52%, respectively.

**Figure-3:**
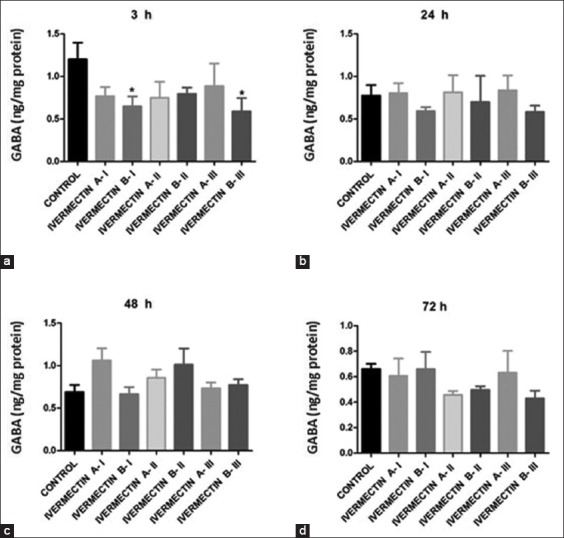
γ-aminobutyric acid levels in *Rhipicephalus* (*Boophilus*) *annulatus* in the control, ivermectin (IVM) A (IVM 10 mg/mL), and IVM B (IVM 10 mg plus clorsulon 100 mg/mL) groups. *p<0.05 versus control, analyzed by two-way ANOVA.

IVM B (I and III groups) showed a significant negative correlation with GABA (ng/mg protein) levels after 3 h of application, whereas no significant changes were observed after 24 h (Figure-3). In contrast, in the IVM A treatment, GABA reached its lowest levels after 72 h.

Tick GABA levels showed a marked drop in the treated groups 3 h after IVM exposure as compared to the control; after 24 h, GABA levels did not show a significant difference in the treated groups; however, there was a noticeable decrease in the IVM B groups.

**Figure-4:**
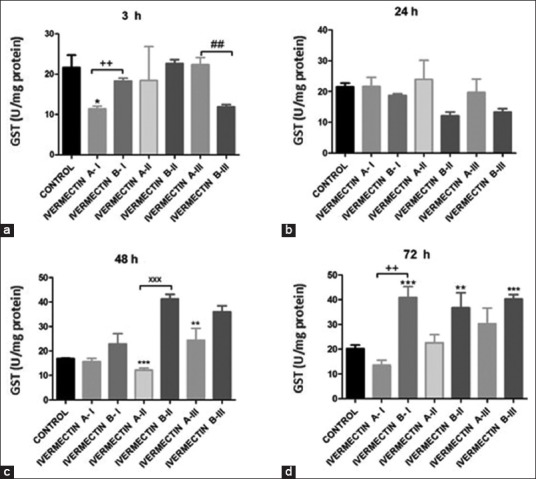
Glutathione S-transferase activities in *Rhipicephalus* (*Boophilus*) *annulatus* of the control, ivermectin (IVM) A (IVM 10 mg/mL), and IVM B (IVM 10 mg plus clorsulon 100 mg/mL) groups. *p<0.05, **p<0.01, and ***p<0.001 versus control, analyzed by two-way ANOVA. ^++^p<0.01 IVM A-I versus IVM B-I; ^xxx^p<0.001 IVM A-II versus IVM B-II, and^##^p<0.01 IVM A-III versus IVM B-III groups by unpaired t-test analysis.

Both IVM A and B upregulated the *R*. (*B*.) *annulatus* antioxidant defense capacity through GST enzyme activity (U/mg protein) in live *R*. (*B*.) *annulatus* in a time-dependent manner (Figure-4). GST activity decreased 3 h after treatment with either IVM A-I or IVM B-III. GST declined after 24 h, followed by a gradual but significant increase after 48 and 72 h in the IVM B-I, II, and III groups.

MDA (nM/mg protein) fluctuated with exposure time to the IVM chemotherapeutics; in the IVM B-II and III groups, MDA levels decreased significantly compared with those in control and IVM A groups, which were markedly lower after 24 h; however, its levels increased in the treated groups after 48 h.

**Figure-5:**
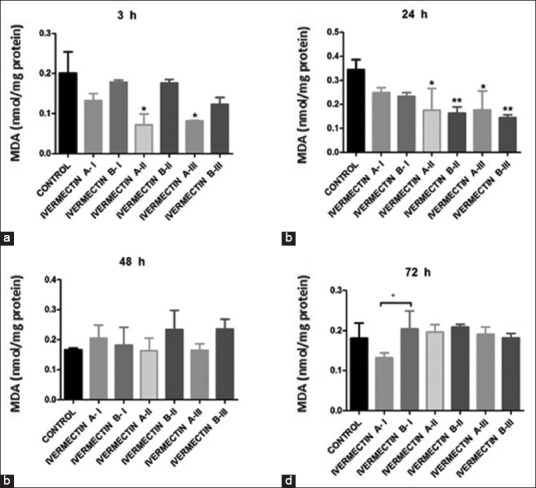
Malondialdehyde levels in *Rhipicephalus* (*Boophilus*) *annulatus* of control, ivermectin (IVM) A (IVM 10 mg/mL), and IVM B (IVM 10 mg plus clorsulon 100 mg/mL) groups. *p<0.05 and **p<0.01 versus control, analyzed by two-way ANOVA. ^+^p<0.05 IVM A-I versus IVM B-I groups by unpaired t-test analysis.

After 3 h, both IVM A-II- and III-treated groups showed a significant drop in MDA levels. Furthermore, IVM B-II and III exhibited a significant change in MDA activity after 24 h. The treated groups showed changes in MDA levels compared with the control group after 48 and 72 h (Figure-5).

## Discussion

The presented study was designed to develop an effective bioassay technique for evaluating *R*. (*B*.) *annulatus* sensitivity to IVM acaricide based on oxidative/antioxidant homeostasis inside the *R*. (*B*.) *annulatus* after IVM exposure. Results revealed that IVM induced mortality and slowed movement (physical observation) of *R*. (*B*.) *annulatus* in a dose-dependent manner, with the highest effect observed in the IVM B-treated groups [28]. Interestingly, IVM was reported to have effectively eradicated cattle tick *R. microplus* [17] and *R*. (*B*.) *annulatus* [3].

GABA is induced either by G protein-coupled receptors or receptors that act as chloride-conducting ligand-gated ion channels [29-32]. GABA-mediated chloride entry into the cells mediates cell membrane hyperpolarization. GABA generates an inhibitory effect on the associated muscles and caused a marked decrease in the frequency of spontaneous action potentials [33,34]. Therefore, GABA and its receptor were considered effective targets for acaricidal drugs for tick population control [35]. Understanding, the acaricide pathway will be important in the development of novel acaricides as well as for the extended control of the cattle tick [36]. A previous study revealed that IVM was an effective anti-parasitic agent against ticks and mostly acted by direct or indirect stimulation of GABA receptors, as GABA has been known to potentiate dopamine-induced fluid secretion in the salivary glands of female ixodid ticks through receptors at which IVM is not an agonist [9]. Therefore, more studies related to the GABA receptors will be required to fully understand the associated mechanism.

GST detoxifies xenobiotics by combining with these foreign chemicals, thereby assisting in their elimination from the body [37]. GST detoxifies heme when the ticks suck an animal’s blood because heme generates reactive oxygen species that lead to the oxidation of lipids, proteins, and DNA [38]. Besides enzymatic functions, GST has a significant role in heme and porphyrin detoxification in invertebrates [39]. This study showed a significant decrease in GST activity at 3 and 24 h after treatment with IVM A-I and IVM B-III due to the prominent GST detoxification effect of IVM on *R*. (*B*.) *annulatus*, accompanied by a significant decrease in MDA levels. The surviving *R*. (*B*.) *annulatus* resisted the effect of IVM on GST levels with time. Thus, the suppression of tick GST action to interrupt their detoxification system and heme metabolism has potential as a tick control strategy [40,41].

In arthropods, ML sensitivity was also associated with amplification in oxidative metabolic pathways [42,43]. This phenomenon was consistent with the fact that tick nourishment and detoxification pathways against foreign chemicals were fundamentally required for the viability of *R*. (*B*.) *annulatus*. The misuse of tick control acaricides, such as IVM, exposed the ticks to a possible dose-dependent non-linearity of IVM in plasma, tissue binding, or both. This allowed for the survival of *R*. (*B*.) *annulatus* population after IVM treatment.

As the less-sensitive *R*. (*B*.) *annulatus* population reported a significant increase in GST activity compared to the sensitive *R*. (*B*.) *annulatus* population, the same causal connection between IVM-counteracting ticks and significant contribution in GST activity seemed logical [44]. The results obtained revealed that IVM showed a potential acaricidal effect on *R*. (*B*.) *annulatus* through inhibition of the detoxification enzyme GST in the initial 24 h of treatment, and it can be seen that IVM B showed higher acaricidal effects than IVM A groups.

More studies on the GST activity of IVM-treated *R*. (*B*.) *annulatus* will be necessary to assist in the recognition of GST behavior in IVM efficacy among tick populations and to provide an understanding the ticks’ possible resistance to IVM treatment among Egyptian herds. Treatment strategies have to be pointed out to keep the impact of selection minimal, while still achieving an appropriate control of the parasite. High IVM treatment frequency (≥4 times per year) may increase the risk of developing resistance to IVM among the *R. microplus* populations. Strategies for early detection of resistance as well as strategies to increase the shelf life of IVM are recommended [45].

## Conclusion

The use of IVM to control *R*. (*B*.) *annulatus* in cattle is expected to increase in Egypt, due to its broad-spectrum, increasingly accessible prices, and lack of effective chemical alternatives. This study recommends further studies on the use of IVM chemotherapeutics to evaluate susceptibility among the *R*. (*B*.) *annulatus* populations and the potential for IVM resistance.

## Authors’ Contributions

HMS, AHE, and AAE designed the study and analyzed the data; AAA, EKA, and GEB contributed to the reagents, materials, and analysis tools; and MMA and YSE contributed to the analysis tools, collected the material, and analyzed the data. All authors read and approved the final manuscript.
